# How Shall We Treat Scarlet Fever?1Read at the annual meeting of the Medical Association of Central New York, June 16, 1893.

**Published:** 1893-09

**Authors:** Frederic W. Bartlett

**Affiliations:** 523 Delaware Avenue; Buffalo, N. Y.


### HOW SHALL WE TREAT SCARLET FEVER?[1]

By FREDERIC W. BARTLETT, M. D., Buffalo, N. Y.

THE interrogatory is rather an unusual way to introduce individual opinions regarding practical proceedings in the treatment of disease, and it signifies, in this case, that the essayist relates, with timidity, his method and results, more with expectation of obtaining information than a conviction that he has a more excellent way than his fellow co-workers. It is, furthermore, not intended, in the brief time allotted to papers, to make any reference to the

1. Read at the annual meeting of the Medical Association of Central New York, June 16, 1893.

etiology or pathology of the disease. Newmarket imports no coals. Briefly, then, I present my plan of dealing with ordinary cases of scarlatina, more especially as regards disinfection, general and local.

Given a case of scarlet fever, the treatment may be defined under the following program:

1. Preparation of the apartment to be occupied by the patient. This should be as isolated from the family as possible, preference being given to an apartment upon upper or attic floor, not communicating with another. Carpets and all other movables, window curtains, mattresses, etc., if the value is to be considered, removed, and plain, wood chairs, old worn sheets, blankets, quilts, etc., substituted. If in the country, oat straw may be used for making the mattress; and in the city, in humble homes, planing mill shavings answer very well. These should be sprinkled with turpentine, solution of carbolic acid, ol. of menth. pip., and spirits of camphor. The bedstead may also be sponged with glycerine, in which some aromatic oil has been dissolved, which will, by its affinity for moisture, always retain any infectious germs. The patient's apparel should be selected from articles whose destruction will be least noticed; and, these preliminaries settled, he should be placed upon the bed which, if he survives, will be his home for two or three weeks. If the rash is perceptible, the first treatment will be friction with the best pulvis sinapis, and this should be repeated every fourth hour, removing excess after each application with a napkin, *moistened* with a solution of bichloride hydrarg., 1-8000 grains. If excess of temperature occur, it should be met by antipyretics. If the throat, on inspection, is found to be inflamed, gargles of boric acid, two teaspoonfuls to the pint of water, may be employed, or the same solution sprayed at frequent intervals over the pharynx. Water may be allowed *ad libitum*. For the protection of the cervical glands, an ointment composed of hydrarg. bin iodide, grains, iii.; lanolin, ℥ii., should be applied from mastoid to mastoid, including the trachea down to the sternum, three or four times daily. The hair, if long, must be shortened, and boys should be as closely clipped as possible, and the head sponged once daily with a 1-8000 solution of hydrarg. bichloride. Milk forms the preferable diet, but fruit, broths, etc., may be substituted. The mustard frictions are to be used for three or four days, or until the rash subsides, the object being to invite the blood, with its contained septic material, to the cutaneous surface, and thus lessen the amount in the viscera and glandular system. These means coöperate with Nature's efforts, and all experience shows that patients with well-developed eruption are far less afflicted with dangerous sequelæ. When the mustard frictions are suspended, the patient is to be sponged morning and evening with warm carbonate soda solution, a half ounce to the quart of water, the applications to be followed by inunctions of olive oil. This is the toilet for ten days or more. After that, sponging once daily with soap suds, particular attention being given to the axillæ, groins, and interstices between the fingers and toes.

The evacuations should be received in glazed vessels, and, perhaps, bichloride solution, 1-4000, sufficient to cover the solid deposits, is preferable to any other. A tablespoonful is sufficient for urinary or liquid motions. These may at once be deposited in flushing water-closets, but otherwise they should be retained longer, and buried far away from the dwelling, or source of water supply. For general disinfection and prophylaxis, sulphur fumigation is, probably, preferable to any other agent. It is best effected by enclosing the powder in a small bag, made from cheese cloth, and shaken in small amount over a hot stove cover, carried through the house on a lifter. In this way it is thoroughly diffused, and the fine dust is promptly ignited.

In place of rags or old linen, the supply of which is often soon exhausted, tissue or closet paper, which has been sprinkled with weak bichloride solution and then dried, may be substituted. This destroys the disease germs, and is readily consumed. The attendant should wear a close-fitting cap, so as to exclude germs, and should occasionally wash the head with boric acid or other disinfecting solution.

As a covering to the floor, roofing-felt, cheap and easy of application, forms the best material. Siding paper will do. An excellent and effective germicide is flame, and this can be utilized by laying strips of old newspapers in a large disused basin, through which a stout wire has been thrust and bent at an angle. Holding the paper in position by some sort of weight, and, by raising and lowering the basin, the flame will consume all floating particles. The care of the patient should be entrusted to not more than two persons, and the garments worn by them, preferably cotton or linen, should occasionally be thoroughly fumigated. Intercourse of the patient with other members of the family should not be permitted before the end of the third week, and the apparel should be thoroughly aseptic.

Among the most useful possibilities of the future, ozonized air may be mentioned. I have had abundant proof, in many years' experience, of its effect upon vegetable forms of life, to which, by common consent, pernicious microbes are restricted.

In addition to antiseptic precautions heretofore described, I cause to be attached, by safety pins to the clothing, over upper sternum, a pad, three by three inches, composed of two or three layers of flannel, and moistened several times daily with a liquid, the formula for which is:

| | | |
|---|---|---|
| ℞.— | Ol. Terebinth. | ℥ s. |
| | Ol. Eucalyptus | ℥ s. |
| | Ol. Menth. Pip. | ℥ s. |
| | Tr. Iodine | ʒ ii. |
| | Chloroform | ℥ s. |
| | Spirits Camphor | ℥ s. |
| | Acid Carbolic | gr. x. |
| | Ol. Olivæ | q. s. fr. |

M. ℥ iv. S.—Add 40 to 60 drops, three or four times daily.

These pads to be worn by all the members of the family. It is specially intended for the children thus far exempted, so as partially, at least, to protect the fauces from the lodgment of infecting germs; and, worn under the outer dress, I find this plan a good one in diphtheria and whooping cough.

*Enteroclysis.*—The great success attending enteroclysis, or flushing the colon, in dysentery, typhoid fever, and diarrhea, has encouraged me to test the same treatment in diphtheria, measles, and scarlet fever. There can be no doubt that the ptomaines and bacilli may find refuge in the convolutions of the intestines, particularly in the colon, and if the special virus may be active for months in clothing, furniture, etc., why not in these fructifying localities, where temperature and culture fluids are so favorable to constant development? There can be no question as to the transmission of the disease by the evacuations, and this not only in the acute stage, but many days after it has passed, and cutaneous desquemation has, apparently, ceased. I adopt the same policy in one condition as the other, and thoroughly irrigate the colon. The patient is placed upon the right side, so as to gain advantage from gravitation in the transverse colon. For a child, from two to ten years old, two to three pints of the solution, according to the following formula, is employed:

℞.—Hydrarg. bichlo. .................... grs. ii.
Aq. Fontana......................... O. ii.

This is modified sometimes for children to half strength, and is usually employed after the eighth day—sooner if there is diarrhea. In all the cases treated, I have never observed a baleful effect from the mercurial, and the good results have been so apparent that I can conscientiously recommend it. Of course, boric acid, tannin, and other agents may be used and found efficient, but when one has been so fortunate as to lose no cases in many years, treated by this special solution, he can well afford to congratulate those who discover a plan, exempt from any possible ill-effect from the germicide used in solution.

A very important precaution in the treatment of scarlet fever is the detection of kidney insufficiency. I provide for this purpose a dropper, a small vial of nitric acid, and a test-tube. Every day for four weeks I direct a test to be made, and if the urine is permanently cloudy, or deposits albumen, I am promptly warned. Generally, if this condition is early detected, it will be found amenable to treatment. If neglected, the probabilities of a temporary or permanent cure are greatly lessened. Among the more efficient means to be employed, is the moist hot pack, in the manner here described.

Prepare the bed by covering the mattress with a rubber blanket; over this a padded quilt or woolen blanket, and fill four to six pint or quart bottles with hot water, tying in the corks with twine beyond the possibility of becoming loosened. Wring a blanket from hot water and wrap the child in it, and place upon the prepared bed. Then pack the bottles two or three on each side of the patient, covering each well, and locating opposite shoulders, hips, and knees. Cover all with a warm, dry blanket or spread, and envelop the head in a napkin which has been dipped in cold water, and which must be frequently renewed. Keep the patient thus perspiring for twelve or more hours, and give water freely. Avoid diuretics, such as digitalis, acet. potash, etc., and give, preferably, calomelas and jalap in appropriate and repeated doses. I recommend, also, when obtainable, poultices over the abdomen, made by bruising the common chickweed, the virtues of which, used in this manner, I accidentally discovered.

I have seen a child with distended scrotum and general anasarca completely relieved in a few hours by this simple remedy. As regards the hot pack, if perspiration is not induced, or, if induced, the patient declines drinks of any kind, it should not be too long continued, nor if the temperature rises. Since I have adopted the disinfecting plan, as heretofore described, I have been quite indifferent as to internal medication. Regulating temperature and diet, and otherwise promoting the comfort of the patient, has seemed to meet all wants.

By pursuing the plan represented in this essay, I have rarely had more than one case in a family, even where there was from eight to twelve children. When the excellent hygienic directions given by the State Board of Health of Michigan, which may be obtained at any time from its secretary, Dr. Henry B. Baker, of Lansing, are faithfully followed, or those lately issued by Dr. Ernest Wende, Health Commissioner of Buffalo, there will be slight risk of the transmission of the disease. When we consider the great direct mortality from scarlet fever, the many sequelæ, such as chronic otitis, with impaired or total loss of hearing, the inauguration, not unfrequently, of acute and fatal nephritis, or its ultimate development in the chronic forms, and other scarcely less important pathological effects, we cannot fail to be impressed with the great responsibility assumed when treating the disease. Indifference or neglect are inexcusable. To be reminded in after years that any of these misfortunes was due to lack of knowledge of the best treatment, medical and hygienical, is a contingency we should strive industriously to avoid.

I desire to briefly summarize the suggestions made in this essay.

1. The hygienic directions in preparing the sick-room.

2. The local cutaneous treatment by dry mustard frictions, followed by bichloride sponging, later by sodii carb. bath and oil inunctions.

3. The breast pad, medicated as described.

4. Enteroclysis, with solutions hydrarg. bichloride 1 to 8,000 or 16,000, varying with the age of the patient.

5. The hygienic essentials as formulated by Drs. Baker and Wende, and boards of health generally.

6. The moral obligations imposed upon the physician, far too frequently ignored, to be faithful to his trust in every detail, upon the intelligent discharge of which such important interests depend.

NOTE.—Having been, so far as I can discover, the originator of the treatment of dysentery, typhoid fever, etc., by intestinal flushing with *germicide* solutions, notably and usually hydrarg. bichloride, my published reports dating back to 1883, and being interrogated often as to the method employed, I herewith define it fully:

Place the patient upon the right side, and so that the back is turned outwardly. Use suitable protectives for mattress.

Use, preferably, an Alpha syringe, or one in which exit tubes are inserted, not fitted by screw attachments.

Dissolve the bichloride in a glazed vessel, never in a metallic one. A half-gallon fruit jar is very good.

Make connection for the rectal tube by cutting off the small bone finish from an ordinary number ten catheter (American) and in an Alpha, push the cut end well back in exit end of syringe. This makes a perfect connection.

Oiling the catheter, insert, at first, partially and gently, and inject fluid slowly. Then, by careful manipulation, the catheter may gradually be introduced its full length. In adults, insist on using and retaining the full quantity, two quarts. With children, permit the outflow. In very irritable children I often induce partial chloroform anesthesia. As soon as the one or two quarts used has been introduced, place the patient upon the slop jar, rather than usual vessel, so as to accommodate the ejected fluids. I have known serious annoyance from limited capacity. On returning the patient to the bed, place on the left side, to obtain gravitation of the contents of the transverse colon.

Treat all cases of colitis, from whatever zymotic cause, in this way, and, if my results are realized, you will hereafter so treat all such cases, even in young children,. Many cases of enterio colitis are cured by one enema.

523 DELAWARE AVENUE.

---

THERE are at the present moment 131 ladies registered as students at the seven French medical faculties. Of this number, ninety-five are Russians, twenty-five French, four Roumanians, two Bulgarians, two Servians, one German, one Turk, and only one English. No one who has studied in Paris can avoid remarking the preponderance of the Muscovite element among these fair aspirants for medical honors.—*Lancet.*

